# The quest for equity in global health is underpinned by neocolonial discourses: A critical discourse analysis

**DOI:** 10.1371/journal.pgph.0004663

**Published:** 2025-06-13

**Authors:** Michelle Amri, Jan Filart, Jesse B. Bump

**Affiliations:** 1 The W. Maurice Young Centre for Applied Ethics, School of Population and Public Health, University of British Columbia, Vancouver, Canada; 2 Faculty of Health Sciences, Simon Fraser University, University Dr W, Burnaby, Canada; 3 Takemi Program in International Health, Harvard T.H. Chan School of Public Health, Boston, Massachusetts, United States of America; 4 Bergen Centre for Ethics and Priority Setting, Department of Global Health and Primary Care, University of Bergen, Bergen, Norway; University of Washington Department of Global Health, UNITED STATES OF AMERICA

## Abstract

Global health, as noted in the emerging decolonizing global health literature, is built on power asymmetries and inequities, is centred on individuals and organizations in the global north, and involves a north to south diffusion of ideas and resources. Despite increasing attention paid to the decolonization of global health, there is no universal understanding of what this entails, or what associated agenda(s) may be. We argue that decolonizing global health is not possible without interrogating its many power asymmetries. In this article we demonstrate one example, using a critical discourse analysis of a tremendously influential document, the final report of the World Health Organization’s Commission on Social Determinants of Health, *Closing the gap in a generation: Health equity through action on the social determinants of health.* This report brought mainstream attention to health inequities and the broader forces that underpin them. We reasoned that a flagship report focused on equity and the social determinants of health would be sensitive to the many power inequities in global health. Our critical discourse analysis reveals normative views that presume inequity, such as Euro-American-centricity and portraying countries of the global south as behind or inferior to those of the global north and requiring support. Also, we find that many country comparisons exclude rich countries, which hides the full extent of global inequity. By drawing attention to the inequities presumed in language, we illuminate the persistence of neocolonial ideas that accept rather than contest unfairness.

## Introduction

The roots of global health lie in tropical medicine [1], an academic specialty concerned primarily with safeguarding the interests of colonial and imperial powers [2] and advancing their ideologies, including religion [3]. Much of global health remains centred on individuals and organizations in the global north [4,5] and “presumes a north to south diffusion of ideas and resources […]” [6]. However, in the last few years, there have been many calls to decolonize global health [4,7] or embrace anti-colonial approaches. Although colonialism is difficult to define, we understand it as a practice of subjugation over people by conquering territory [8], or the direct political and/or economic control by one nation over another. European colonialism has evolved into Euro-American neocolonialism, as Kwame Nkrumah wrote in 1965 [9], referring to the perpetuation of metropolitan control even after political decolonization, mainly by economic means. For the purposes of our paper, we use neocolonialism to indicate the present-day consequences of colonialism and/or various ongoing manifestations of the power inequalities of that period. As such, neocolonialism includes complicity between “neocolonizers” and the “neocolonized” that is expressed by the “new world economic order” or neoliberalism, which has also been called “coloniality” [10].

Despite ongoing attention to decolonizing global health, there is no accepted uniform or universal understanding of what this entails or what its associated agenda(s) may be [11]. For instance, a convening by the editorial board of *Global Health Research and Policy* stated that to “fully decolonize global health, systemic reforms must be taken that target the fundamental assumptions of global health: does investment in global health bring socioeconomic development, or is it the other way around?” [12]. However, Büyüm et al. note that “Decolonising global health advances an agenda of repoliticising and rehistoricising health through a paradigm shift, a leadership shift and a knowledge shift” [13]. And further, Abimbola and Pai note that “to decolonise global health is to remove all forms of supremacy within all spaces of global health practice, within countries, between countries, and at the global level” [4]. As Finkel et al. note, suggestions for addressing decolonization vary greatly [14], but in our opinion, none of these views are incompatible with our focus on neocolonialism and its inequities.

In keeping with Abimbola and Pai’s view, we argue that removing supremacy is not possible without interrogating the language and discourse of global health. Although there have been historical works that discuss how global health is shaped by colonial patterns and inequities [15,16], we are not aware of any previous examination of how these patterns shape the details of technical policy work in health. We pursue this line of investigation by analyzing one of the most influential reports by one of global health’s largest players, the final report of the World Health Organization’s (WHO) Commission on Social Determinants of Health (CSDH), *Closing the gap in a generation: Health equity through action on the social determinants of health* [17].

**Fig 1:**
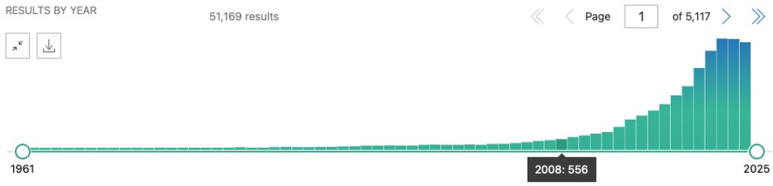
Temporal depiction of PubMed hits for “social determinants of health” (captured on November 26, 2024).

**Fig 2:**
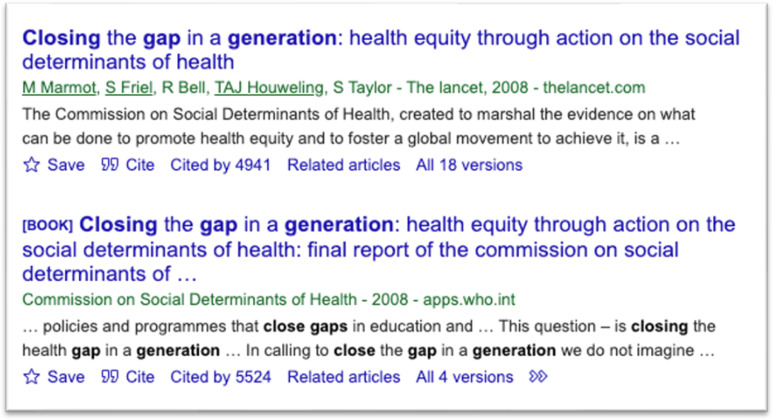
Google scholar entries for “Closing the gap in a generation” (captured on November 26, 2024).

The CSDH’s final report sought “to marshal the evidence on what can be done to promote health equity, and to foster a global movement to achieve it” (p. 3) and has been tremendously influential in advancing attention to the social determinants of health. (The WHO categorizes the social determinants of health as: income and social protection; education; unemployment and job insecurity; working life conditions; food insecurity; housing, basic amenities and the environment; early childhood development; social inclusion and non-discrimination; structural conflict; and access to affordable health services of decent quality [18]). First, the report has promoted the view that systematic differences are “inequitable and avoidable, with socioeconomic, political, and historical causes” [19]. Second, it has contributed to global awareness around health equity, as opposed to more narrow focus on acute problems [20]. This increased attention to the social determinants of health may be assessed by tracking the phrase in PubMed-indexed publications. The first use of this phrase is in 1961, but 46,747 of the 51,169 results (91.4%) appear in 2008 and onwards, corresponding with the date of publication of the CSDH’s final report (Fig 1). Third, it has spurred several initiatives, such as the WHO’s Urban Health Equity Assessment and Response Tool [21]. Fourth, its influence on research. According to Google Scholar, the report and a related paper in *The Lancet* have been cited more than 10,000 times in the decade and a half since they were published (Fig 2). As there have been no similar reports since its publication, we can have greater confidence about the influence of the document whose language we analyze.

## Methods

Our primary method is critical discourse analysis (CDA), a multidisciplinary, problem-oriented approach that seeks to study social phenomena [22]. CDA has no single definition or universally agreed process, but in general it treats language as a form of evidence and analyzes it within a social context. We analyzed *Closing the gap in a generation* line-by-line in its entirety (256 pages) to develop inductive codes (CDA has been applied to other WHO texts, as well [23,24]). All text passages and graphical elements were examined this way, including tables, figures, and images. Although many CDAs focus on coding words, our approach to also code images and figures aligns with scholarship by Maposa, who notes the exclusion of Mauritius or Seychelles in maps of Africa reveals a limited spatial conceptualization of the continent [25]. Coding was conducted by MA using NVivo12 software and resulted in the main codes of: “development” (e.g., how is this term is used?), “global south vs. north” (e.g., how are countries discussed and what direction of impact is mentioned or implied?), “poor”, and uses of language that indicated a presumption, such as “surprisingly.” Codes were approached inclusively, meaning surrounding text was included as part of the code where relevant, to ensure context and meaning was not lost. These codes were then read and analyzed for overarching themes, as presented in the manuscript. The results were reviewed and agreed upon by the authors. No approval from a research ethics board was required, as *Closing the gap in a generation* is publicly available.

## Results

This analysis identifies many statements that imply certain normative positions, including depictions of the global south as subaltern or “behind”; “othering” of countries of the global south; disregard for colonial histories; presumptions that global south countries need support; and a reliance on economic conceptions of development. In addition to these normative views, we examined how countries are compared and grouped. These are discussed in the following sections.

### Normative views

#### Global south as subaltern or “behind”.

We repeatedly found the normative view is that the global south is subaltern [26] or “behind” the global north. This has consequences for perpetuating neocoloniality. For instance, through stating “Improved working conditions in high-income countries, hard won over many years of organized action and regulation, are *sorely lacking* in many middle- and low-income countries” (p. 5, emphasis added) presumes that workers in the global south are unwilling or unable to organize. This example and nine others, as noted in S1 Table, express the view that the global south is behind, typically implying causes that would be domestic to those countries. An exception to this pattern appears on page 135 and notes that “Absolute levels of alcohol-related disease and disability are as high in the poorest countries of Africa and America as in Western Europe and North America” (p. 135). This unusual statement of similarity uses a negative indicator. We observe that this statement could be presented in a neutral manner. For example, “countries in Africa, America, Western Europe, and North America face similarly high absolute levels of alcohol-related disease and disability” or flipped to present successes in the global south (e.g., describing successful policies or initiatives to curb alcohol consumption).

In addition to this view that countries of the global south are subaltern, passages also convey a sense of disbelief of countries of the global south can be similar to those in the global north, and in some instances, attribute shortcomings to personal failings. For example, “Within countries, the differences in life chances are dramatic and are seen in all countries – *even the richest*” (p. 26, emphasis added) and others noted in S2 Table. Similarly, other passages convey surprise that successes have been realized in the global south which we report in S3 Table. A discussion of Sri Lanka’s intersectoral action for health highlighted failures that were “attributed to the existing structures of decision, the lack of capacity to identify intersectoral links and become proactive on them, and the prevailing administrative culture” (p. 113). Despite the challenges associated with successfully implementing intersectoral and multisectoral approaches to health experienced worldwide [27–30], the highlighted challenges largely place the onus on individuals’ inability (e.g., lack of capacity and non-proactive behaviour).

#### “Othering” of countries of the global south.

“Othering” entails seeing those unlike oneself in an “outgroup” who are inferior [31]. Therefore, we understand “othering” in the exclusionary sense, meaning using “power within relationships for domination and subordination” [32]. There are statements which reflect a certain difference or “othering” process between countries of the global south and north. This can be quite subtle, such as through stating “infectious diseases and undernutrition will continue in *particular* regions and groups around the world” (p. 4, emphasis added). Or this can be quite explicit, such as in stating “Some countries remain profoundly disempowered through lack of resources relative to others” (p. 157). It is important to ponder what such statements are in relation to. What does “particular regions” or “some countries” refer to? Who decided that these countries are the “other”? What implications do such classifications have?

#### Disregard for colonial histories.

Countries of the global south are also not just seen as subaltern but their past histories, including resource extraction, are not fulsomely examined. The report states that “increasingly the nature of the health problems rich and poor countries have to solve are converging” (page 3). Although it is true that certain conditions are common in both “rich” and “poor” countries, this formulation disregards grander problems, such as inequity (despite the prior sentence pointing to the importance of social and economic policies by stating that “Social and economic policies have a determining impact on whether a child can grow and develop to its full potential and live a flourishing life, or whether its life will be blighted”) and the determinants of said problems, which are arguably more important to address for sustained improvements to health. This leads to the question: was inequity never a problem all countries needed to address? Or similarly, by stating that “Low-income countries should strive to progressive realization of universal coverage, starting with the most vulnerable” (p. 55), it is worth asking why past histories are not being comprehensively considered and further, why this advice is specific to low-income countries when universal health coverage is an ongoing process in all countries? Of the three instances in which colonization is mentioned, two are in reference to Indigenous populations (pp. 36 and 160) and the last is with respect to countries being under colonial rule when the United Nations Security Council was established (p. 169).

Further to this stance of glossing over past histories to treat countries of the global south and north universally autonomously, the “Note from the chair” indicates that “[The report] provides analysis of social determinants of health and concrete examples of types of action that have proven effective in improving health and health equity in countries at all levels of socioeconomic development” and in the executive summary, “Importantly, the focus of attention embraces countries at all levels of income and development: the global South and North. Health equity is an issue within all our countries and is affected signiﬁcantly by the global economic and political system”. Making such a statement implies that delineation by countries of different socioeconomic development is perhaps not necessary, given how pervasive inequities are. This is also supported through noting that “While evidence on the effects of [redistributive welfare] systems comes mainly from high-income countries, where data are available and policies are in place, it does show the potential effect of social protection policies more widely” (p. 85). Ultimately, this perspective challenges discourses present in the report, discussed in depth under the “country comparisons” section below. Referring to the statement in the “Note from the chair” and that these global challenges transcend levels of socioeconomic development, should the discussion of global problems also not transcend past specific country income-level groupings? It seems that such comparisons are used to glorify the progress of countries of the global north. There is an assumption that countries of the global north and south should have different disease burdens, as if this classification itself acts as a determinant of health.

#### Countries of the global south need support.

We also criticize the presumption of helplessness and denial of agency that underlies the sense that global south countries *require* support. For example,

*“Low- and middle-income countries are not likely, in the near future, to be able to provide all the funds needed to create an entirely healthy living environment. Funding from more afﬂuent countries will be required to support the plans made by peoples and governments in less afﬂuent countries”* (p. 63).

Or expressed in terms of reliance on donors, such as through stating “Given the drastic limits on domestic ﬁnancing in low-income countries, ofﬁcial ﬁnancial ﬂows in the form of aid and debt relief are critical to addressing dramatic global health inequities” (p. 120), or through the Commission’s recommendation that “Donors, multilateral agencies, and Member States build and strengthen national capacity for progressive taxation (…) particularly building institutional capacity in low-income settings” (p. 123). This demonstrates a perception of a hierarchy and the strength of influence of the global north, in addition to promoting colonial models of aid. However, there are little mentions of factors that lead to demise or who is responsible for this “hierarchy”, as per above.

#### Development focused on economy.

And not only is the global south seen as subaltern generally, but also economically inferior. Despite statements like “There is growing demand for a new approach to social development – one that moves beyond an overriding focus on economic growth to look at building well-being through the combined effects of growth and empowerment” (p. 28), this perspective reflects a larger view of “development” being economic-centric. This is evidenced through employing language like “affluent”, “poor” and “rich” countries, emphasis on economic development, and securing means for “development”—each showcased in Table 1. Further, clear distinctions are drawn about different countries around “development” based on common employment conditions through statements such as the following:

**Table 1:** Term or concept demonstrating that “development” is economic-centric and associated sample quote.

Term or concept	Sample quote
affluent	“…it is still true that the more affluent flourish and the less affluent do not” [p. 26]
rich	“This is critical for poor countries in which […]” [p.7]
emphasis on economic development	“Building up universal social protection systems will require changes in the global economy and national economic policies, allowing all countries to reach the level of development at which this is feasible and sustainable in the long term” [p. 89]
securing means for “development”	“Whatever may be the merits of any single proposal, taxing ﬁnancial transactions to raise revenue for development is now widely regarded as both feasible and appropriate” [p. 125]

*“A starting point is state provision of a quantum of jobs. This has different implications for countries at different levels of development. In low-wage settings such as India, (…). In many OECD countries, where most of the workforce is formal and there is relatively low unemployment (…)”* (p. 78).

This focus on economic development, although it may seem appropriate, does not reflect “(…) that good and equitable health do not depend on a relatively high level of national wealth” (p. 33), “that the MDG [Millennium Development Goal] for reduction in poverty implies attention to the distribution of income not just economic growth” (p. 38), that “Growth in global wealth and knowledge has not translated, either, into increased global health equity” (p. 167), or other non-economic considerations. For instance, there is recognition of the need for “(…) the empowerment of people, communities, and countries that currently do not have their fair share” (p. 23), but through the report’s differentiation between “social” and “economic development” (p. 35), this perhaps implies that “development” used alone, does not naturally entail social development. With this consideration, should “development” not also discuss how these groups (i.e., people, communities, and countries) can or have been empowered?

### Country comparisons

#### Exclusion of countries of the global north.

Larger comparisons are often made across countries of different classifications (i.e., low-income, lower-middle-income, and upper-middle-income countries), but fail to include high-income countries or countries of the global north. For instance, Fig 2.1 (p. 29) graphs infant mortality rates between countries and within countries by mother’s education. We believe it would not be difficult to make comparisons to rich countries to better demonstrate inequities — particularly because this figure is in the section “Health Inequity in All Countries” (p. 29). In other instances, these differences are hidden by comparing only global north countries, such as across OECD countries. For instance, Figure 11.4 (p. 126) compares taxation in East Asia with sub-Saharan Africa. This comparison is presented to draw attention to how “East Asia saw strong growth and improvements in health throughout the period, while sub-Saharan Africa experienced large-scale stagnation and, in some cases, decline” (p. 124). However, given the report’s broader argument that direct taxation (e.g., income, property) is preferential to indirect taxation (e.g., trade, sales), would it be better to compare sub-Saharan Africa to countries where direct taxation is the norm? Is drawing a comparison to East Asia rooted in seeking to demonstrate actions that are “within reach” given similar economic positions of countries within these regions? Through failing to compare to the most obvious countries with such direct taxation systems in place, does this comparison further this idea that countries of the global south are operating in their own dimension, one that is behind that of the global north?

#### Country groupings may transcend geography.

Countries are grouped in a manner that may transcend geography to arguably fit a narrative around what countries can aspire to and hide the full extent of inequity globally. Figure 6.2 (p. 62) depicts percentage changes in road-traffic deaths since 1987. Countries are grouped into the categories of “Central and Eastern Europe”, “Latin/Central America and the Caribbean”, “Middle East and North Africa”, and “highly motorized countries”. Aforementioned groups are geographical regions with the exception of the latter. The latter is comprised of “North America, Australia, New Zealand, Japan, and Western Europe”. It is an interesting choice to group such countries when they could be mapped in similar geographical categories (e.g., “North America”, “Oceania”). This is further exasperated through noting that the decline in deaths in highly motorized countries “offers hope for other countries where motorization is on a steep upward slope” (p. 62), as this choice of language evokes an emotion of aspiration rather than unequivocally stating the evidence around such public policies.

Unusual country groupings are also presented in Figure 7.1 (p. 72), where the graph depicts deaths from workplace exposure to dangerous substances. Understandably, China is mapped independently as it has the highest rate, however, why is it also mapped by region, as there are the categories of “Middle East Crescent” and “Latin America and the Caribbean”, and economic system, as there are “Formerly Socialist Economies” and “Established Market Economies”. Another instance of this appears in Figure 11.1 (p. 121), which maps the proportion of tariffs in total revenue by region from 1980 to 1998. Although groupings mapped are “South Asia”, “Latin America & Caribbean”, “East Asia & Pacific”, “Sub-Saharan Africa”, “Middle East & North Africa”, “Europe & Central Asia”, and “Industrialized Economies”, there is no “North America” grouping which is arguably included in the “Industrialized Economies” category, meaning there is data available.

## Discussion

The preceding results demonstrate several normative views, which are the: perception of the global south as subaltern or “behind” the global north, “othering” of countries of the global south, disregard for colonial histories, positioning of countries of the global south as needing support, and focus on the economy in “development”. In addition to these normative stances, it was also observed that country comparisons often exclude countries of the global north and that country groupings may transcend geography.

We believe it is essential to question the Euro-American-centric stance and “othering” of countries of the global south. This Euro-American-centricity is associated with a perception of countries of the global south as subaltern countries of the global north. Why is it that this placement exists? Who benefits from this status quo and does this inhibit action? There are hints of this understanding that come through the report. For instance, stating that “In low-income countries, competitive advantage is heavily dependent on low labour costs, and this may be compromised if provision of a regularly updated decent living wage becomes a statutory requirement” (p. 78) reflects this understanding that the “success” of countries of the global north is *dependent* on the “failures” of countries of the global south. This aligns with the work of Assis who notes that “From the perspective of coloniality, the old colonial hierarchies, which were grouped into the European versus non-European relationship, continued to be rooted and entangled in the international division of labor” [33]. Is it enough to note that “Under conditions of market integration, poor countries in particular have been losing important forms of public revenue (…) raises issues regarding the fairness in global ﬁnance of public resources in low-income countries” (p. 87), or is there a need to move beyond it simply being raised as an issue? Who stands to gain from disrupting the status quo and are they incentivized to act?

At a more subtle level, the maps employed, see Figure 6.1 (p. 61), whereby certain countries and regions are granted more “weight” by taking up more space visually — a problem of the Mercator projection — results in misconceptions and distortions in our ideas [34]. Further, something seemingly as simple as capitalizing “North” and “South” in “global north” and “global south” (e.g., “Women’s health centres were established in many countries of the North and also in some countries of the South” [p. 150]), relays an idea of a definition (i.e., proper noun). However, the “global south” is not a definition determined by geography, but rather, it is determined by the Bruntland line [35,36]. Therefore, through a lack of capitalization in writing the “global south”, the non-physical determination of this space is reinforced [35]. For this reason, we elected to keep “global south” and “global north” as lowercase to combat the narrative that these are physically determined spaces (the original capitalization was used in direct quotes). And although some scholars seek to resist binaries (e.g., global north and global south) to help produce alternative ways of knowing [37], given that “differentiation emerges at every scale, shaped by how residents of any place, living prosperously or precariously, are differently positioned within and through the trans-local processes” [38], this does not seem to be the case in the CSDH’s report. These, seemingly minor aspects, provide a foundation for remaining views, that should not be taken at face value.

This analysis also demonstrates how countries of the global south are positioned as needing support. Although global efforts for global challenges are arguably beneficial, through the language employed, a positioning of countries of the global south as mere beneficiary not only removes agency, but it places undue heroism on countries of the global north and donors. In seeking to move away from the colonial history of the roots of global health, reconsideration is needed for the emphasis on aid and donors. These ideas may also be linked to the finding around the heightened focus on the economy in “development”. Perhaps, if discussions on “development” and associated focus on the economy are replaced with strength-based approaches focused on discussing successes, discourses can better deflect slants of global south countries as passive beneficiaries, promote country agency, and move away from perpetuating neocolonial views.

Language such as “countries at risk of conﬂict” (p. 123) does not come with clarification around why there is conflict or who is responsible, or similarly for “poorest of the poor” (p. 31), who this entails and what structures and processes led to this outcome. Such language can neutralize discussions and potentially abstract responsibility from countries of the global north. Statements like “Yet global inequities in power inﬂuence the ability of poor countries in particular to enact policies that are optimal for child development” (p. 51) are a positive step, but still remove responsibility from actors. Although we understand the political constraints of the WHO and the broader United Nations, we believe the report should have more deeply examined extractive processes or the interaction of countries, much of which is still unfair.

The CSDH, chaired by Sir Michael Marmot, was constituted to include Commissioners with expertise in science, public health, policymaking, and social change, with responsibilities in advocacy and political leadership [39]. Although most Commissioners are from the global south, the report writing team was composed of five scholars with global north affiliations, who also comprised most of the secretariat team. Members of Knowledge Networks collated and synthesized evidence for the report. As seen in the report’s “Acknowledgements”, attendees of the Commission’s Vancouver meeting were highlighted as key participants in providing review and commentary for the report. It is possible that the processes in which the report was produced may have unintentionally promoted harmful discursive patterns for global health, in which othering and binary discourses were utilized. Although problematic, it is possible this was rooted in broader structural issues at the time, which recent movements are trying to break.

It should ultimately be noted that we examined the work of this Commission from a reconfigured perspective thanks to progress made in discourses surrounding decolonizing global health and social equity across almost two decades since its publication. Historical context must be taken into consideration when analyzing such texts. Documents produced by an international health authority such as this holds, communicates, and reproduces power. As such, a CDA approach helps reveal how views have evolved over time, as well as the future direction global health discourse ought to take.

### Strengths and limitations

This CDA was undertaken with the view that this is a first step in uncovering prevalent discourses in global health. This study does not seek to present an all-encompassing analysis into the field, but rather, provide an initial glimpse into such discourses through investigating the CSDH’s *Closing the gap in a generation*. Therefore, it is plausible and likely that there are more neocolonial discourses in the field that need to be interrogated and combatted. This methodology can and should be applied to other normative technical documents to examine and potentially expose whether similar discourses are present across different agencies and sectors.

We further note that we analyzed the English text of the CSDH’s *Closing the gap in a generation*, although it is available in seven additional languages. This approach to analyze the English report was due to English being our primary language of operation. However, we suspect that analyses of the additional reports will unveil additional nuances. By operating in English, we are also unable to draw on criticisms in the field not expressed in English, serving as a limitation to our broader discussion.

## Conclusions

By drawing attention to the inequities presumed in language, we illuminate the persistence of neocolonial ideas that accept rather than contest unfairness. For instance, by accepting an inherent Euro-American-centric stance and othering of countries of the global south, we accept unfairness. Not only do we accept unfairness in language or ideas, but unfairness in policy and practice perpetuated in global health. If the CSDH’s final report is any indication of other seminal policy and technical documents—which we presume it is—it entails that global health is operating within an implicit and explicit neocolonial discourse. Policy and practice and being developed, implemented, and evaluated within this neocolonial frame, which will have major repercussions on who is directing policy agendas, resources, relationship dynamics between stakeholders, and many other aspects. Such norms influence outputs and outcomes, which can also be less impactful, disregard certain segments of the population, and just generally, perpetuate inequity.

Through investigating shortfalls and fallacies, we narrow in on opportunities for improvement, both with respect to the work of the WHO and more broadly. By shifting global health away from its colonial foundations, we can move toward a more just and equitable praxis. We should strive for praxis that seeks redress for historic injustices and reforms the unjust political economy of global power, which is one of the most significant sources of ongoing inequity.

## Supporting information

S1 Table Samples passages alluding to the global south as subaltern. (DOCX)

S2 Table Sample passages conveying a sense of disbelief at how challenges are not confined to the global south or need to clarify such. (DOCX)

S3 Table Sample passages conveying a sense of disbelief at global south successes. (DOCX)

## Abbreviations

CDA: Critical discourse analysis
CSDH: Commission on Social Determinants of Health
WHO: World Health Organization
