# Supplementary material for: The quest for equity in global health is underpinned by neocolonial discourses: A critical discourse analysis
Source: PLOS Glob Public Health. 2025 Jun 13;5(6):e0004663. doi: 10.1371/journal.pgph.0004663 (PMC12165370; doi:10.1371/journal.pgph.0004663)
Supplement: S1 Table — (DOCX) [file pgph.0004663.s001.docx]

# Table **1.** Samples passages alluding to the global south as subaltern

| **Page number** | **Sample passage** |
| --- | --- |
| p. 7 | “While limited institutional infrastructure and ﬁnancial capacity remains an important barrier in many countries, experience across the world shows that it is feasible to start creating social protection systems, even in low-income countries” |
| p. 31 | In rich countries, low socioeconomic position means poor education, lack of amenities, unemployment and job insecurity, poor working conditions, and unsafe neighbourhoods, with their consequent impact on family life. These all apply to the socially disadvantaged in low-income countries in addition to the considerable burden of material deprivation and vulnerability to natural disasters. So these dimensions of social disadvantage – that the health of the worst off in high-income countries is, in a few dramatic cases, worse than average health in some lower-income countries (Table 2.1) – are important for health.” |
| p. 37 | “For any country – arguably most pressingly for countries with low incomes – economic growth brings the possibility of great beneﬁt” |
| p. 81 | “In many of the poorest countries, it is also likely that a large proportion of entrepreneurs in the informal sector will have minimal levels of education or literacy, severely limiting their ability to conform to regulatory requirements” |
| p. 85 | “Countries with more generous social protection systems tend to have better population health outcomes, at least across high-income countries for which evidence is available” |
| p. 87 | “While limited institutional capacity remains an important barrier, it is feasible even for poor countries to start building social protection programmes, as shown by experience across the world” |
| p. 131 | “Experience shows the real potential of such mechanisms even in low-income contexts, despite informational and political challenges” |
| p. 136 | “The institutionalization of health equity impact assessment is clearly still in its infancy – and presents real issues in terms of required technical skills and institutional capacity in many countries, especially those with low and middle incomes” |
| p. 136-137 | “Although ﬂexibilities are formally written into the Trade-related Aspects of Intellectual Property Rights (TRIPS) agreements, governments – particularly in many low- and middle-income countries with limited technical and institutional capacity – have in many cases derived only limited beneﬁt from such ﬂexibilities, and have in others been encouraged not to use them at all” |
