# Supplementary material for: The quest for equity in global health is underpinned by neocolonial discourses: A critical discourse analysis
Source: PLOS Glob Public Health. 2025 Jun 13;5(6):e0004663. doi: 10.1371/journal.pgph.0004663 (PMC12165370; doi:10.1371/journal.pgph.0004663)
Supplement: S2 Table — (DOCX) [file pgph.0004663.s002.docx]

# Table 2. Sample passages conveying a sense of disbelief at how challenges are not confined to the global south or need to clarify such

| **Page number** | **Sample passage** |
| --- | --- |
| p. 30 | “The social gradient is not confined to poorer countries” |
| p. 31 | “We need to be concerned with both material deprivation – the poor material conditions of the 40% of the world’s population that live on US$ 2/day or less – and the social gradient in health that affects people in rich and poor countries alike” |
| p. 46 | “To be sure, [health inequities] are bigger in scale in some countries than in others but they are remarkably widespread” |
| p. 50 | “Even in high-income countries such as the United Kingdom, infant mortality is higher among disadvantaged groups” |
| p. 60 | “But slums are not only a problem of low- and middle-income countries; 6% of urban dwellers in high-income regions live in slums” |
| p. 64 | “Many cities in rich and poor countries alike are facing a crisis in the availability of, and access to, affordable quality housing” |
| p. 64 | “It is shocking that in an economically rich country such as the Republic of Ireland, a remarkable 17% of households are fuel poor” |
| p. 65 | “Although outwardly afﬂuent, the city of Torquay in the south of England has pockets of deprivation” |
| p. 86 | “In low- and middle-income countries, the proportion of older people is growing even faster than in high-income countries” |
| p. 90 | “While in many countries there may be a tendency to target social protection programmes to the most deprived, there are strong arguments for setting up universal protection systems, even in poor countries” |
| p. 94 | “Over half a million women die each year during pregnancy or delivery or shortly thereafter, virtually all in low- and middle-income countries (WHO, 2005b). Lack of access to and utilization of adequate maternity care is a key factor in this appalling statistic. In many countries, both poor and rich, costs of health care can lead to disastrous impoverishment” |
| p. 94 | “Health care is inequitably distributed around the world. The pattern of inequity in utilization is pronounced in low- and middle-income countries, but inequity is prevalent in high-income settings too” |
| p. 103 | “There are circumstances, in rich and poor country contexts, in which health-care users pay fees directly for services” |
| p. 121 | “Many countries have not been able to replace these losses with other sources of public revenues or taxation. As a consequence, a majority of low-income countries have seen a net decline in overall public revenues (however, for many low income countries, this trend has been arrested or reversed since 1998). Middle-income countries have fared  slightly better, but in general trade liberalization has translated into a reduced capacity of national governments to support public expenditures in health, education, and other sectors (Baunsgaard & Keen, 2005; Glenday, 2006). High-income countries, with already well-established taxation systems and existing public infrastructures, have been able to move away from tariff revenues with minimal loss in ﬁscal capacity. But increasing intensity of global tax competition (real or perceived) has also had negative effects on national ﬁscal capacity, even in high-income countries (Tanzi, 2001; Tanzi, 2002; Tanzi, 2004; Tanzi, 2005).” |
| p. 134 | “Even in the richest economies there are segments of the labour force where conditions are very poor” |
| p. 149 | “The main goal of gender mainstreaming in Sweden is to tackle the structural roots of gender inequity in society at large. Having such a goal is far from being the norm even within the EU” |
| p. 153 | “Even in poor countries, childcare facilities are feasible (…)” |
| p. 189 | “BOX 16.13: POOR HEALTH LITERACY – ALSO A PROBLEM IN RICH COUNTRIES” |
