# Supplementary material for: The quest for equity in global health is underpinned by neocolonial discourses: A critical discourse analysis
Source: PLOS Glob Public Health. 2025 Jun 13;5(6):e0004663. doi: 10.1371/journal.pgph.0004663 (PMC12165370; doi:10.1371/journal.pgph.0004663)
Supplement: S3 Table — (DOCX) [file pgph.0004663.s003.docx]

# Table 3. Sample passages conveying a sense of disbelief at global south successes

| **Page number** | **Sample passage** |
| --- | --- |
| p. 87 | “Whereas trade liberalization and tax competition can erode the ability and/or willingness of governments to strengthen universal social protection systems, this is not universally the case. Indeed, some of the East Asian countries strengthened their social protection policies when faced with economic downturn” |
| p. 91-92 | “Existing schemes in countries such as Bolivia, Lesotho, Namibia, and Nepal show that creating a basic social protection system is administratively and practically feasible in low- and middle-income countries, despite obvious challenges” |
| p. 96 | “Virtually all high-income countries organize their health-care systems around the principle of universal coverage (combining mechanisms for health ﬁnancing and service provision). But commitment to universal care is not limited to high-income countries. Thailand, for example, has shown leadership and success” |
| p. 100 | “In Asia, public spending on health was redistributive in 10 of 11 countries, while four others achieved a pro-poor, or even, distribution of health beneﬁts (O’Donnell et al., 2007; O’Donnell et al., 2005). In ﬁve of seven Latin American countries, public spending on health was either proportionally distributed across rich and poor groups or weighted to the poor (PAHO, 2001). Even in Africa, where concern has been expressed about inequity of public health-care expenditure, spending was found to be redistributive in all of the 30 countries studied (Chu et al., 2004)” |
| p. 101 | “The Ghana example (Box 9.9) has shown signs of dependence, but also shows how bold moves towards universal pre-payment are possible” |
